# Forearm Basilic Vein Transposition: A Single-Centre Experience

**DOI:** 10.7759/cureus.40129

**Published:** 2023-06-08

**Authors:** Umair A Khan, Talha Kareem, Muhammad Uneeb, Omer Ehsan, Hassan Wyne

**Affiliations:** 1 Vascular Surgery, Shifa International Hospital, Islamabad, PAK; 2 General Surgery, Shifa International Hospital, Islamabad, PAK; 3 Urology, Multan Institute of Kidney Diseases, Multan, PAK

**Keywords:** arteriovenous fistula, vascular access procedures, arteriovenous access for renal dialysis, chronic kidney disease (ckd), basilic vein transposition

## Abstract

Introduction: Forearm basilic vein transposition (FBVT) is a viable alternative for arteriovenous grafts (AVGs) and can be used as secondary vascular access as well, as it allows for the use of veins that are remote from the arterial source of inflow. FBVT involves two main steps: first, the basilic vein is dissected from its original location; and second, the basilic vein is transposed to a subcutaneous tunnel on the volar aspect of the forearm and anastomosed to a suitable artery, usually the radial or ulnar artery.

Objective: This paper aims to present a series of FBVT cases performed at our hospital and present it as a viable option for secondary vascular access. We also aim to review the available literature relating to FBVT fistula in terms of surgical technique, patency rates, maturation time, and one-year outcome, and to establish a comparison with our clinical experience.

Materials and methods: This is a retrospective descriptive case series. The data were collected from online medical records, and patients were contacted by telephone to make a follow-up visit. For a review of the literature, a search was done on PubMed for articles containing the following keywords in either the title or the abstract: basilic, transposition, fistula, and forearm. Similarly, a search was done on Google Scholar for articles with the following words in the title: basilic, transposition, and forearm. The data are expressed as mean and standard deviation. Statistical analysis was done using SPSS 26.0 software (IBM Corp., Armonk, NY).

Conclusion: The primary patency rate of FBVT in our study makes it a suitable solution to opt for before moving to AVGs. FBVT should be considered before moving more proximally in patients with inadequate forearm cephalic veins.

## Introduction

Forearm basilic vein transposition (FBVT) is a surgical technique that involves creating an arteriovenous fistula (AVF) between a superficial basilic vein and a radial or ulnar artery in the forearm. FBVT can be a viable alternative for arteriovenous grafts (AVGs) and can be used as secondary vascular access as well, as it allows for the use of veins that are remote from the arterial source of inflow [1]. According to the National Kidney Foundation's Kidney Disease Outcomes Quality Initiative (KDOQI) guidelines, patients should have fistulae placed starting distally with a snuff box fistula. If the distal AVFs fail, the access can be moved up proximally. Autogenous AVFs are preferred over synthetic grafts because they have lower rates of infection, thrombosis, and intervention, and higher rates of patency and adequacy [2].

Basilic vein transposition in the forearm involves two main steps: first, the basilic vein is dissected from its original location; and second, the basilic vein is transposed to a subcutaneous tunnel on the volar aspect of the forearm and anastomosed to a suitable artery, usually the radial or ulnar artery. The transposition of the vein facilitates needle cannulation by dialysis personnel and improves patient comfort during haemodialysis (HD). The procedure can be performed under local or regional anaesthesia, with or without sedation, depending on patient preference and comorbidities.

Objective

There are not enough studies available in Pakistan evaluating FBVT as a viable dialysis access option. In fact, there is only a single case report published in 2023 assessing the patency and feasibility of FBVT [3]. This paper aims to present a series of FBVT cases performed at our hospital and present it as a viable option for secondary vascular access. We also aim to review the available literature relating to FBVT fistula in terms of surgical technique, patency rates, maturation time, and one-year outcome, and to establish a comparison with our clinical experience.

## Materials and methods

This is a retrospective descriptive case series. The data were collected from online medical records and patients were contacted by telephone to make a follow-up visit.

Between January 2017 and March 2023, 14 patients had FBVT at our hospital. All patients underwent clinical evaluation with a duplex scan. Radial or ulnar artery diameter > 2 mm and basilic vein diameter > 2 mm were taken as the inclusion criteria. FBVT was not performed in patients with a basilic vein diameter of less than 2 mm or a thickened radial or ulnar artery with a diameter of less than 2 mm.

For a review of the literature, a search was done on PubMed for articles containing the following keywords in either the title or the abstract: basilic, transposition, fistula, and forearm. Similarly, a search was done on Google Scholar for articles with the following words in the title: basilic, transposition, and forearm. The results were then reviewed. Duplicates and irrelevant articles were excluded.

The data are expressed as mean and standard deviation. Statistical analysis was done using SPSS 26.0 software (IBM Corp., Armonk, NY).

Surgical technique

**Figure 1:**
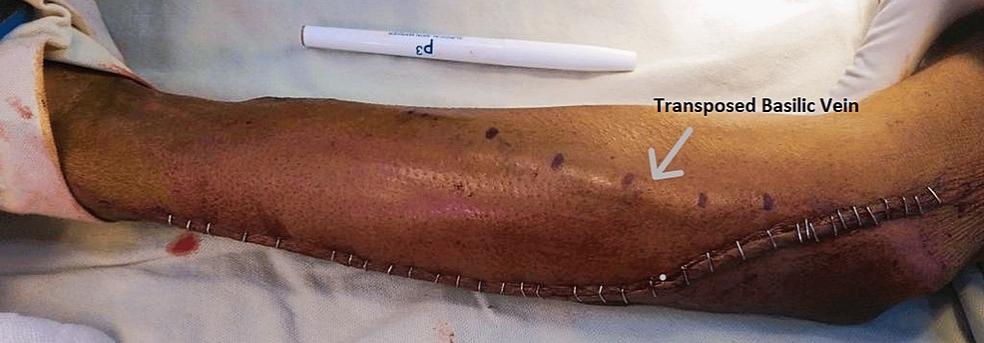
Basilic vein transposition in the forearm

All the procedures were carried out using a combination of local anaesthesia and intravenous sedation using midazolam. Once the entire arm was prepped and draped, a longitudinal incision was made directly over the skin mark of the mapped basilic vein to perform a basilic vein transposition in the forearm. The incision began at the medial aspect of the antecubital fossa and then proceeded distally towards the wrist. The tributaries were tied with Polyglactin 3-0 suture. Once the vein was dissected it was wrapped with a saline-soaked sponge. Another separate skin incision was made over the radial artery above the wrist, and after dissection of the radial, a longitudinal arteriotomy was performed. The artery was flushed with heparinized saline before clamping proximally and distally. A subcutaneous tunnel in the volar aspect of the forearm was created, and the vein was passed through the tunnel after marking it with a skin marker and continuous inflation using heparinized saline. An end-to-side anastomosis was then performed between the radial artery and basilic vein using polypropylene 6/0. Finally, the clamps were removed, and the propagating thrill overlying the transposed basilic vein was checked. The wound was closed in reverse order with the subcutaneous tissue closed with Polyglactin 3-0 suture and the skin closed using either a 4-0 Monocryl suture or skin staplers (Figure 1).

## Results

A total of 14 patients underwent FBVT. Eight patients were male and six were female. All the patients were hypertensive, and nine patients were diabetic. The mean age was 65.57 ± 8.62 years (Table 1).

**Table 1:** Demographic details

n	14
Age	65.57 ± 8.62 years
Diabetics	09 (64.3%)
Hypertensives	14 (100%)
Males	08 (57.1%)
Females	06 (43.9%)

The time taken for the maturation of AVF was 49.85 ± 5.66 days. The AVF maturation time refers to the number of days following its formation after which a successful dialysis can be performed through this AVF. The primary patency was 12.67 ± 5.83 months and the primary assisted patency was 21.33 ± 4.61 months. Three patients needed angioplasty to salvage the AVF. Eleven of the patients had previous dialysis access in the form of a central venous catheter (CVC) or another AVF. Three patients had no previous dialysis access and were pre-dialysis.

The patients had a follow-up six months and one year after the procedure. There were no wound site complications. One patient was lost to follow-up. At six-month follow-up, 86% (11/13) of fistulas were working. After one year, 77% (10/13) were patent. Two patients eventually had access failure, and in one patient, the fistula failed to mature and needed alternative dialysis access (Table 2).

**Table 2:** Experience of our centre with FBVT AVF: arteriovenous fistula; FBVT: forearm basilic vein transposition.

n	14
Time for AVF maturation	49.85 ± 5.66 days
Primary patency	12.67 ± 5.83 months
Patients with previous dialysis access	11 (78.6%)
Pre-dialysis	03 (21.4%)
Patients needing intervention to salvage AVF	03 (21.4%)
Patency at six months	11/13 (86%)
Patency at one year	10/13 (77%)
Access failure	02 (14.3)
Maturation failure	01 (7.1%)

## Discussion

The earliest evidence of FBVT extends back to 1976 when Zincke and Aguilo used a basilic vein loop to salvage the radio-cephalic (RC) AVF that got thrombosed. The proximal part of the cephalic vein was ligated, and an anastomosis was made between the distal cephalic vein and the basilic vein [4]. Based on this study, Lindstedt and Lindergård, in 1980, conducted a case series of FBVT. They tunnelled the basilic vein in the forearm and anastomosed it to the radial artery. The primary failure rate in these patients was 24%. In 17 of these patients, the fistula was used for dialysis for a duration of one to 30 months [5].

In recent times, studies involving FBVT have shown better results. While primary autogenous RC fistulae are preferred, the need for secondary and tertiary options is on the rise due to the increasing number of elderly patients requiring HD.

The primary patency of FBVT has been compared with the traditional methods and although there is a significant improvement in the primary patency of FBVT, the traditional RC fistula, however, remains the first choice. Several studies have reported favourable outcomes of basilic vein transposition in the forearm for HD access. Silva et al. [1] reported a series of 89 patients who underwent this procedure as primary access. They found that 91% of these access procedures matured sufficiently to support sustained HD, with a one-year patency rate of 84%.

Gameel et al. in 2012 reported the primary patency of FBVT to be 52% at one-year follow-up. This was significantly less than the primary patency of RC AVF but better than AVG, hence favouring the autogenous approach [6]. Vadivelu et al. in 2018 published a case series of 15 patients with FBVT and the primary patency at one year was 73.3%. Similarly, primary patency rates at one, two, and three years were 77%, 62%, and 21%, respectively, according to Zielinski et al. [7]. Results, however, have not always been consistent and according to Son et al., the primary patency of FBVT at 12 months was 41.5%. The primary patency though lower than most of the other studies was still higher than the one observed for AVG [8]. Weaver et al. in 2019 also showed a lower primary patency rate for FBVT with only 46.9% of the fistulas patent after one year [9]. Glowinski et al. published a series of 30 patients who had a failed RC AVF and had an FBVT as secondary vascular access. All fistulas matured and had the primary patency of 93% at one-year follow-up [10]. One of the recent studies conducted by Uzun et al. yielded similar results with a fistula maturation time of 45.2 ± 10.7 days and a primary patency of 90.5% at one-year follow-up [11]. Jairath et al. also had convincing results. He created FBVT using the skip incision technique and conventional techniques. More than 80% of the basilic vein transpositions matured at 12 weeks and had primary patency of around 70% at one-year follow-up in both groups [12].

In our experience, the primary patency was at 77% in one year, which was better than several studies. The primary patency in months is less than that of the study carried out by Zielinski et al.; however, the primary assisted patency is 21.33 ± 4.61 months. Eleven (78.5%) patients in our study had previous dialysis access and seven of them had two or more than two previous failed accesses. The maturation time was 49.85 ± 5.66 days, which was comparable to the previously conducted studies (Table 3).

**Table 3:** Comparing various parameters of FBVT in different studies FBVT: forearm basilic vein transposition.

Authors	Number of patients	Primary patency (months)	Maturation time (days)	Maturation failure	Primary patency at one year	Patients with previous dialysis access	Access failure
Gameel et al. (2012) [6]	25			4	52%	20 (80%)	
Zielinski et al. (2018) [7]	24	23 ± 14.4	39 ± 7.0	1	77%		1
Son et al. (2010) [8]	34			5	41.5%		1
Weaver et al. (2019) [9]	34			4	46.9%	24 (70.6%)	4
Gormus et al. (2003) [13]	10			0	90%	2 (20%)	1
Glowinski et al. (2014) [2]	30			0	93%	30 (100%)	3
Weyde et al. (2008) [14]	27			5	70.4%		
Uzun et al. (2019) [10]	21		45.2 ± 10.7		90.5%		
Silva et al. (1997) [1]	89			8	84%	0	10
Our experience	14	12.67 ± 5.83	49.85 ± 5.66	1	77%	11 (78.5%)	3

Autogenous AVFs are a reliable way to gain access for HD with a low risk of infection and thrombosis. Using native vessels to create AVFs has several advantages over HD access grafts, including lower infection rates, a favourable cost/benefit ratio, and longer-term use with a lower incidence of intimal hyperplasia [15-17].

The dialysis population has been on the rise due to factors such as reduced availability of renal transplants, increased patient survival on HD, and a growing number of patients referred for renal replacements [18]. As a result, finding appropriate access for patients with end-stage renal failure is becoming a more significant issue. The algorithm for dialysis access places distal access as a priority, and therefore, FBVT can serve as a valuable tool to deal with the growing population of patients on renal replacement therapy.

To prevent early-term failure of created AVFs, proper preoperative care for end-stage renal disease, education, and preoperative imaging are essential.

The limitations of our study include a small patient cohort and the nonrandomized nature of our analysis. However, this does not change that FBVT should be considered as a comparable option in appropriately selected patients with inadequate forearm cephalic veins prior to moving to the proximal upper extremity and should always be considered before moving to AVGs.

## Conclusions

The primary patency rate of FBVT in our study makes it a very suitable solution to opt for before moving to AVGs. In fact, FBVT should be considered before moving more proximally in patients with inadequate forearm cephalic veins.

## References

[1] Silva MB Jr, Hobson RW 2nd, Pappas PJ (1997). Vein transposition in the forearm for autogenous hemodialysis access. J Vasc Surg.

[2] Lok CE, Huber TS, Lee T (2020). KDOQI clinical practice guideline for vascular access: 2019 update. Am J Kidney Dis.

[3] Riaz T, Rehman ZU, Riaz SA (2023). Radio basilic transposition arteriovenous fistula : an effective but less frequently used haemodialysis vascular access - a case report. J Pak Med Assoc.

[4] Zincke H, Aguilo JJ (1976). Basilic vein swingover for creation of arteriovenous fistula of forearm for hemodialysis. Urology.

[5] Lindstedt E, Lindergård B (1980). Transposition of the basilic vein in the forearm for the construction of haemodialysis arteriovenous fistula. Scand J Urol Nephrol.

[6] Gameel AM, Samir AM, Sorour WA (2012). The use of basilic vein transposition in the forearm as an alternative autogenous hemodialysis access. Ain Shams Med J.

[7] Zielinski M, Inston N, Krasinski Z, Gabriel M, Oszkinis G (2018). The forearm basilic vein looped transposition fistula as a tertiary option for upper limb vascular access. J Vasc Access.

[8] Son HJ, Min SK, Min SI, Park YJ, Ha J, Kim SJ (2010). Evaluation of the efficacy of the forearm basilic vein transposition arteriovenous fistula. J Vasc Surg.

[9] Weaver ML, Holscher CM, Sorber R, Arnold MW, Lum YW, Reifsnyder T (2019). Comparison of forearm versus upper arm basilic transposition arteriovenous fistulas demonstrates equivalent satisfactory patency. J Vasc Surg.

[10] Glowinski J, Glowinska I, Malyszko J, Gacko M (2014). Basilic vein transposition in the forearm for secondary arteriovenous fistula. Angiology.

[11] Uzun HA, Çiçek ÖF, Seren M (2019). Transposition of basilic vein in forearm for arteriovenous fistula creation: our mid-term results. Turk Gogus Kalp Damar Cerrahisi Derg.

[12] Jairath A, Singh A, Sabnis R, Ganpule A, Desai M (2017). Minimally invasive basilic vein transposition in the arm or forearm for autogenous haemodialysis access: a less morbid alternative to the conventional technique. Arab J Urol.

[13] Gormus N, Ozergin U, Durgut K, Yuksek T, Solak H (2003). Comparison of autologous basilic vein transpositions between forearm and upper arm regions. Ann Vasc Surg.

[14] Weyde W, Letachowicz W, Krajewska M (2008). Arteriovenous fistula reconstruction in patients with kidney allograft failure. Clin Transplant.

[15] Thwaites SE, Holt SG, Yii MK (2021). Inferiority of arteriovenous grafts, in comparison to autogenous fistulas, is underestimated by standard survival measures alone. ANZ J Surg.

[16] Park J, Kim J, Hwang S (2019). Arteriovenous graft patency outcomes and prognostic factors. Vascular.

[17] Mousa AY, Patterson W, Abu-Halimah S (2013). Patency in arteriovenous grafts in hemodialysis patients. Vasc Endovascular Surg.

[18] Romagnani P, Remuzzi G, Glassock R (2017). Chronic kidney disease. Nat Rev Dis Primers.

